# Patterns of caesarean-section delivery in Addis Ababa, Ethiopia

**DOI:** 10.4102/phcfm.v8i2.953

**Published:** 2016-07-08

**Authors:** Yibeltal T. Bayou, Yohana J.S. Mashalla, Gloria Thupayagale-Tshweneagae

**Affiliations:** 1 Department of Health Studies, University of South Africa, South Africa

## Abstract

**Setting:**

The study was conducted in Addis Ababa, the capital city of Ethiopia. Specifically, it was conducted in all healthcare facilities offering maternity and obstetric services.

**Objective:**

The objective of the study was to explore the patterns of caesarean-section (CS) delivery in Addis Ababa.

**Methods:**

A cross-sectional survey was carried out between December 2013 and January 2014. The population for the study were women aged between 15 and 19 years of age who had given birth in the last 1–3 years before the date of data collection. The Census and Survey Processing System software was used for data capturing and analysing both descriptive and inferential statistics using Statistical Package for Social Sciences version 20.0.

**Results:**

Amongst the 835 women who delivered at health facilities, 19.2% had given birth by CS. The prevalence of CS based on medical indication was 91.3%. However, 6.9% of CS performed had no medical indication. Private health facilities performed more CSs than public health facilities, 41.1% and 11.7% respectfully. CS was high amongst women of higher socio-economic standing.

**Conclusion:**

Overall, CS deliveries rate in Ethiopia is above the rate recommended by the World Health Organisation. Because socio-economic factors influence CS delivery, governments should play a key role in regulating performance of CSs in private institutions.

## Introduction

About 800 women die from pregnancy or childbirth-related complications around the world every day.^1^ Skilled birth attendance is one of the packages of intervention for improving maternal and child health and is one of the MDG indicators to track national effort towards safe motherhood.^2,3^ The use of technology in urban settings for antenatal care (ANC) and delivery care is a common practice both in developing and developed nations. Ultrasound scans during pregnancy, delivery care in high-tech-equipped medical centres or hospitals and use of caesarean section (CS) are the most prominent features. These services can be obtained based on either medical indications or client’s preference to use them.

CS can be life-saving to both the mother and the foetus by preventing poor obstetric outcomes.^4^ However, there is a growing concern on the increasing percentage of the procedure of live births globally.^5,6^ The risks and costs associated with caesarean deliveries are significant, especially where there was no medical indication. Evidence shows that caesarean delivery and maternal death are significantly and positively associated.^7^

According to the 2011 Ethiopia Demographic and Health Survey the rate of CS (22%) in Addis Ababa was far more than the 10% – 15% rate recommended by WHO.^4^ The recommended limit has recently been backed up by the results from 137 countries.^8^ Further research shows that CS rates beyond 15% are considered medically unjustified or unnecessary, with negligible benefits for most mothers, and yet costly and unequally distributed amongst the population.^8,9^

Every pregnant woman in Ethiopia has the right to information about her health; discuss her concerns, thoughts and worries; know in advance about any planned procedure to be performed; privacy; confidentiality; and express her views about the services she receives.^10^ To fulfil these rights, in 2010 the government of Ethiopia developed an Obstetrics Management Protocol based on WHO’s goal-oriented model.^11^ The protocol focuses on a limited set of essential antenatal, delivery, postnatal and newborn care services and prescribed statements about indications in the use of procedures such as CS and ultrasound scanning.^10^

According to the delivery protocol, ‘caesarean-section is performed when safe vaginal delivery is either not feasible (absolute) or would impose undue risks to the mother and/or foetus (relative)’. The protocol states that appropriate indications, presence of a trained provider and appropriate equipment and facilities are the prerequisites for CS. It enforces the provider to explain to the client or relatives about the procedure and to seek informed consent. The protocol also has details of the possible complications of the procedure and it states that maternal mortality is higher after CS than after vaginal delivery. Whilst the protocol is silent about inappropriate CS that can be initiated either by the mother or the care provider without any medical or obstetric indications, it cautions about the possible inappropriate and excessive reliance on technology or procedures and ultimately increasing healthcare costs.^10^ The objectives of the study were to explore the pattern of CS delivery in Addis Ababa.

## Methods and materials

### Study population

The target population for the study were all women aged 15–49 years living in Addis Ababa, the capital city of Ethiopia. To be included in the study, women should have experienced at least one birth in the last 1–3 years before data collection.

### Sampling

The study used a stratified, two-stage cluster design. Because Addis Ababa is entirely urban, stratification was achieved using the sub-cities (10 strata). In the first stage, 30 sample points known as enumeration areas (EAs) were selected independently from all the strata with Probability Proportional to Size of households in each stratum using the 2007 Population and Housing Census data. A new household listing was not conducted for the study, but the random sample selection was carried out using the number of households identified per EA during the 2011 Ethiopia Demographic Health Survey. In the second stage, 906 households were selected with Proportional to Size of households in each EA. The final valid sample size was 901 because of non-response rates. Proportional to Size is a special and efficient method in multistage cluster sampling.^12^ The sampling frame for the first stage was the lists of clusters (EAs) per stratum and for the second stage the lists of households in each EA. Households were the sampling units for the study.

### Data collection

Data were collected from December 2013 to January 2014. A questionnaire was developed by the researchers to assess adequacy of ANC amongst residents in Addis Ababa, Ethiopia. The questionnaire asked women about their most recent births, and a list of questions were also asked to elicit the adequacy of ANC amongst study participants. Demographic and socio-economic information were also included in the questionnaire. Before administration of the questionnaire, it was pilot tested with 15 women who had similar characteristics with the study population but amongst those outside the selected EAs who were not included in the final sample. The findings from the pilot were used to finalise the collection tool. The questionnaire was also translated into Amharic, the local language mainly used in Addis Ababa.

### Description of independent variables

The independent variables were selected based on a modified version of the Behavioural Model of Health Services. This model distinguishes three sets of factors related to healthcare-seeking behaviour of individuals, namely the predisposing, enabling and need factors.^13^ Hence, a number of potential predictor variables were used to examine and explain variations in the adequacy of ANC. Age, number of living children, current marital status and pregnancy intention related to the last childbirth, social structure variables such as education, occupation and ethnicity were considered at individual and household levels. As regards to pregnancy intention, women were asked about their recent birth, whether they wanted it then, wanted later or did not want to have any more children at all. In the analyses, pregnancy intention for the last birth was further defined as a dichotomy variable: intended for births wanted by then versus unintended for either mistimed or unwanted by then. Women’s education was defined as the highest level of schooling attended, regardless of whether the woman completed the level. Educational status was categorised as no education, primary education and secondary or higher education. Mother’s occupational status was categorised as employed and unemployed.

Individual and family resource indicator variables including health insurance and wealth quintile were included. Those who visited the health facility for ANC were asked whether they had an organisation or agency that either partially or fully covered their expenses, and responses were grouped as ‘yes’ or ‘no’. The relative economic status of the households was determined indirectly through the creation of a wealth index. In the analysis, the construction of wealth index was carried out using principal components analysis via a collection of indicators representing durable goods owned by the household, materials used in construction of the home, water and sanitation facilities and size of the home. In the grouping of the wealth status, after obtaining the wealth quintiles, the 879 sample size was classified into five categories of approximately equal numbers ranging from the least advantaged (first quartile or lowest class) to the most advantaged (fifth quintile highest class).

A community resource variable (type of resident) was used in the analysis. Type of resident was categorised based on the five indicators developed by the United Nation Human Settlements Programme.^14,17^ Access to improved water, access to improved sanitation, sufficient living area, durability of housing and secure tenure (housing tenure) were used in the construction of type of resident. According to the United Nation Human Settlements Programme, a household is categorised as non-slum if all of the above five indicators are fulfilled; otherwise, the household was considered slum. Hence, for the study, 837 of the respondents were found to be slum residents.

A woman meeting at least one of the following criteria was classified as having a history of high-risk pregnancy, that is, nulliparous with 35 years or older; more than four previous births; and history of spontaneous abortion, known high blood pressure, diabetes or epilepsy.^15,18^

Finally, ANC was defined as overall adequate if the woman had her first antenatal visit within the first 12 weeks, had at least four antenatal visits and had received 12 basic component ANC service contents at least once in the last pregnancy period as recommended by the National Obstetrics Protocol; otherwise, ANC was considered inadequate.

### Outcome variable, caesarean section

The outcome variable for the study was the mode of delivery for the last birth categorised as vaginal delivery regardless of whether instrument was used or not versus CS delivery.

### Data analysis

In the study, data were captured using the Census and Survey Processing System software and were analysed for both descriptive and inferential statistics using the Statistical Package for Social Sciences version 20.0.^16,20^ Bivariate (chi-square tests) were applied on the descriptive part of the analyses. Logistic regression modelling was undertaken to examine the net effects of set of explanatory variables over the outcome variables, and the odds ratios (OR) were adjusted for all other variables. The outcome variable, mode of delivery, was dichotomised with ‘1’ being CS and ‘0’ being vaginal delivery. For the study, *p*-value < 0.05 was considered as statistically significant at 95% confidence interval.

## Ethical consideration

Ethical clearance was obtained from and the study was approved by the National Research Ethics Review Committee of the Ministry of Science and Technology, Ethiopia.

## Results

Amongst the 835 women who delivered at healthcare facilities, 19.2% of them gave birth by CS. The community-level CS delivery rate amongst all the respondents was 18.0%. Table 1 shows that medical indication was the reason for 91.3% of CSs. The service provider’s influence in the absence of any medical indication and the mother’s request in the absence of medical justification accounted for 1.9% and 6.9% of the reasons for CSs, respectively. Amongst those who preferred CS without medical indication, more than half of them believe that CS is safe for the mother or the child. About 36.4% of them demanded the procedure to avoid labour pain. Nearly two-thirds of the CS clients (65.8%) were not informed by the service providers about the consequences of the procedure.

**TABLE 1:** Reason for caesarean section delivery amongst mothers in Addis Ababa, January 2014.

Causes and information	Frequency	Percentage
**Reason for caesarean section**^a^		
Medical indication	145	91.2
Mother’s request	11	6.9
Service provider’s influence without medical indication	3	1.9
Missing	2	1.2

**Total**	**161**	**100.0**
**Reason for mother’s request**		
To avoid labour pain	4	36.4
Safe for the mother or child^b^	6	54.5
Other	1	9.1
**Told about consequences of caesarean section**		
Yes	55	34.2
No	106	65.8

**Total**	**161**	**100.0**

*Source*: Current Research Findings 2015

a The denominator excludes missing values;

b Only one woman believes safe for the child.

### Disparities in caesarean section delivery

Table 2 presents the disparities of CS delivery across different variables. Statistically significant disparities were observed between CS delivery and the different socio-economic, demographic and other variables.

**TABLE 2:** Disparities in mode of delivery by demographic and socio-economic variables, Addis Ababa, January 2014.

Social structure	CS delivery
**Mother’s educational status; *p* = 0.000**	
No education	9.3
Primary education	13.9
Secondary education	22.2
Tertiary education	33.6
**Mother’s occupation; *p* = 0.203**	
Unemployed	18.2
Employed	22.2
**Ethnicity; *p* = 0.546**	
Amhara	20.3
Guragie	17.2
Oromo	18.8
Tigrie	25.9
Others	15.4
**Healthcare system**	
**Place of delivery; *p* = 0.000**	
Public facility	11.7
Private facility	41.1
**Adequacy of ANC; *p* = 0.001**	
Inadequate	17.6
Adequate	32.6
**High-risk pregnancy; *p* = 0.014**	
Yes	25.5
No	17.5

*Source*: Current study Findings 2015

ANC, antenatal care; CS, caesarean section.

*, Chi-square *p*-value.

Overall, CS delivery rate was much higher than the recommended rate, particularly amongst those with a higher socio-economic status (27.6%); women belonging to the high-wealth quintile household (20.2%); clients of private healthcare facilities (41.1%); women aged 30–49 years (25.1%); currently married women (21.6%); those with intended last pregnancy (20.8); women with secondary (22.2%) and tertiary (33.6%) level of education; women who had health insurance coverage (30.4%); and those who had adequate ANC for the last birth (32.6%).

### Factors associated with caesarean-section delivery

Table 3 shows the unadjusted and the adjusted odds of CS delivery as an outcome variable. Binary logistic regression was run for both ratios. The model included only those women who delivered at healthcare facilities. All the socio-economic, demographic and healthcare variables that showed statistically significant association with the outcome variable (CS = 1, Vaginal delivery = 0) at the significance level of *p* < 0.05 were included in the adjusted binary logistic regression. The latter helps to control possible confounding factors and to see the net effect of each independent variable on the outcome variable whilst controlling all other explanatory variables.

**TABLE 3:** Factors associated with caesarean section delivery (OR with 95% CI), Addis Ababa, 2014.

Variables	COR (95% CI)	AOR (95% CI)
**Age group**		
15–24	1	1
25–29	2.11 (1.25, 3.56)*	1.93 (1.03, 3.62)**
30–49	2.92 (1.73, 4.93)***	2.56 (1.36, 4.93)*
**Marital status**		
Currently married	1	1
Not married (other)	0.50 (0.31, 0.79)*	0.88 (0.52, 1.48)
**Mother’s pregnancy intention**		
Unintended	1	1
Intended	1.60 (1.04, 2.47)**	1.25 (0.73, 2.16)
**Mother’s educational status**		
No education	1	1
Primary education	1.58 (0.74, 3.35)	1.88 (0.74, 4.78)
Secondary and above	3.42 (1.66, 7.03)*	3.10 (1.26, 7.64)**
**Type of resident**		
Slum	1	1
Non-slum	2.16 (1.51, 3.08)***	1.80 (1.17, 2.77)*
**Place of ANC visit**		
Public facilities	1	1
Private facilities	0.99 (0.46, 2.14)**	1.36 (0.71, 2.61)
**High-risk pregnancy**				
Yes	1	1
No	0.61 (0.41, 0.90)**	0.60 (0.38, 0.97)**
**Overall adequacy of ANC**		
Inadequate	-	-
Adequate	2.24 (1.34, 3.75)*	1.83 (1.01, 3.29)**
**Health insurance**		
Yes	1	1
No	0.51 (0.30, 0.88)**	0.82 (0.39, 1.73)

*Source*: Current study findings 2015

ANC, antenatal care; AOR, adjusted odds ratio; CI, confidence interval; COR, crude odds ratio; OR, odds ratio.

* *p* < 0.01;

** *p* < 0.05;

*** *p* < 0.001

Age, educational status, type of residence, history of high-risk pregnancy and adequacy of ANC showed consistent statistically significant associations with CS delivery between the crude and adjusted ORs.

Compared with young mothers ages 15–24 years, older mothers aged 30–49 years had greater odds of CS delivery (OR = 2.56). Women with education at the secondary level and above and non-slum residents were 3.10 and 1.80 times more likely to have CS delivery compared with those with no formal education and slum residents, respectively. History of high-risk pregnancy is positively associated with CS delivery (conversely, OR = 1.67). Women who received overall adequate ANC were more likely to have CS delivery compared to those with inadequate ANC (OR = 1.83). Mother’s current marital status, pregnancy intention, health insurance coverage and place of ANC visit did not show consistent significant association between the unadjusted and adjusted ORs.

## Discussion

In the study, CS delivery rate was examined in relation to the recommended standards. Generally, the procedure was overused in the Ethiopian metropolitan, Addis Ababa. World Health Organization^4^ reported that higher than 10% – 15% rates of CS are not justifiable anywhere. However, reports show that there is an alleged overuse of the procedure in many parts of the world.^8,15^ Results of the study also show that the CS rate of 19.2% in Addis Ababa was much higher than the recommended 10% – 15% rate, suggesting that there is overuse of CS delivery in the studied population. The findings of the study show that CS is higher in Addis Ababa than the reported rates in other urban areas, including Africa and Asia,^17,18,19^ and nearly as high as the average CS rate (20%) in the developed nations.^20^

Many factors influence the use of CS in urban areas. CS has been found to be more frequent amongst nulliparous women, older women, women with high-risk pregnancy and women with better educational and economic status.^5,17,19^ In the current study, after controlling for selected demographic, socio-economic and healthcare factors, the odds of CS delivery were higher for older women, highly educated (secondary or above) women, high-risk pregnant women and those who received adequate ANC.

Maternal age is often indicated as a proxy for healthcare-seeking behaviour. Some evidences show that older women are more likely to use healthcare services, including CS delivery.^21,22^ In the study, women older than 30 years are more likely to have CS delivery compared to younger ones (aged 15–24). This finding agrees with studies conducted in India and Vietnam, where those aged 30 years and above are at least two times more likely to have CS.^17,19^ Their accumulated experience in using health services gives older women more confidence in decision-making towards healthcare. They might also be told by health workers during ANC visits that older age is a risk factor.^23,24^ Older women also have more chance of a prior CS, which leads them to high chance of another CS.^6^

Higher rates of CS in urban areas are associated with the availability of technologically for/and advanced obstetric services, high rates of maternal healthcare utilisation and availability of private healthcare facilities, amongst others.^17,25^ As there are variations between urban and rural areas, such gaps are also commonly observed amongst different groups of the community within urban areas like the poor and the affluent. In the current study, the affluent were nearly two times more likely to have CS birth as they can afford the costly CS delivery.^9^ A study in India showed that economic status and woman’s financial autonomy are associated with institutional delivery.^26^

In the current study, the healthcare variable which has statistically significant association with CS delivery was the adequacy of ANC as a composite indicator for timing, number of antenatal visits and content of services received during ANC visits. Women who received adequate ANC were more likely to have CS delivery compared to those with inadequate ANC. A study in Vietnam shows that overall adequacy was positively associated with CS delivery in rural areas but the urban model did not show any significant association.^19^

## Conclusion

The study describes the current level and pattern of CS delivery in the study area together with associated risk factors to the procedure. The study shows that there is high use of CS especially amongst women with a high social and economic standing, better education, and those who gave birth at private healthcare facilities. CSs performed without medical indications were significant; hence, clear guidelines for CS practices on maternal request should be included in the national Obstetrics Management Protocol. Women need more information about the risks and benefits of CS birth. They should also be educated that use of expensive and exhaustive investigation services does not necessarily imply good quality services.

Future studies need to examine the attitude of service providers and their influence on the growing CS delivery rate. In-depth investigations on the effect of CS delivery on maternal and child health outcomes should be carried out in the study area.

In interpreting the study’s findings, it is advisable to consider some of the limitations of the study. The cross-sectional nature of the data does not allow making causal inferences about the relationship between adequacy of ANC and delivery care and the risk factors. It is important to keep in mind that the analysed data include information reported by mothers only from last pregnancies or childbirths. The study did not collect data about the views and practices of service providers related to quality of services and use of CS delivery. The study was also totally limited to the capital city, and findings might not reflect the situation in the rest of the country.
